# NNTs and NNHs: handle with care

**DOI:** 10.3399/bjgp17X689797

**Published:** 2017-03

**Authors:** Adrian A Root, Liam Smeeth

**Affiliations:** Department of Noncommunicable Disease Epidemiology, London School of Hygiene and Tropical Medicine, London.; Department of Non-communicable Disease Epidemiology, London School of Hygiene and Tropical Medicine, London.

Best medical practice should be informed by research evidence. However, translating study findings into clinical decisions is not straightforward. Ratio measures of effect such as the risk ratio are most commonly reported, but tell us little about absolute effects. For example, taking a statin reduces your risk of vascular events by about 20% a year across a wide range of baseline vascular risk.^1^ If your initial 10-year risk of such an event was 1% then taking a statin would reduce this risk by 0.2%, whereas if your initial risk was 20%, the reduction would be 4%. This shows how the absolute risk difference can be more clinically informative.

The number needed to treat (NNT) statistic was introduced over 30 years ago and has gained popularity as a useful measure to help clinicians understand research findings.^2^ Abstract probabilities are converted into a more tangible quantity: number of patients treated. In the above statins example, the NNT for 10 years to avert one vascular event would be 500 among the low-risk group, but 25 in the high-risk group.

NNT might appear to be an attractive measure to the busy clinician wishing to condense the results of a study into a single summary figure, but relying on it in isolation can be misleading. It is often given a concrete interpretation: ‘50 patients need to be treated to prevent one adverse outcome’. This is incomplete. A more accurate interpretation would be ‘if 50 patients with a similar risk to the study population were treated for the same time as the study population then, on average, one fewer would experience an adverse outcome compared with 50 patients treated in the same way as the study control population during the study period’.

This might seem slightly fastidious but the differences are crucial.

## NNT IS ONLY A STATISTIC

The language of NNT strongly suggests cause and effect: for every 50 people I treat one will be saved from harm. This might be appropriate if the treatment effect is derived from large, well-conducted randomised controlled trials but could be a dangerous assumption for many studies.

## NNT DEPENDS ON THE STUDY CONTEXT

NNT is specific to not only a single treatment, but also a specific study population and time frame. The NNT for statin treatment was 500 for the low-risk patient in the example above but 25 for the high-risk patient per year, and lower still for longer treatment periods. Therefore, to apply an NNT to your patient the first question should be, ‘How similar is my patient to the patients in the study?’ This is especially important when NNTs for different alternative treatments are compared. If the study populations have different baseline risks, then the NNTs cannot be compared. Similarly, care must be taken in generating NNTs from meta-analyses where the individual studies might have very different baseline risks.

## NNT IS AN AVERAGE ASSOCIATED WITH UNCERTAINTY

NNT might sound like a concrete number but in fact it is an average measure that, like all statistics, is associated with uncertainty. We should therefore expect it to be quoted along with a measure of its uncertainty. Usually, this is expressed in terms of a confidence interval (CI); this is a plausible range of values given the study findings. Although CIs can be generated for the NNT, they can be confusing because an ineffective treatment will have an NNT of infinity.

For example, 6% (95% CI = −3% to 15%) more people reported a good recovery from ankle sprain at 3 months after receiving physiotherapy and this absolute risk difference translates to an NNT of 16.7 (number needed to treat to harm [NNTH] 33.3 to ∞ to number needed to treat to benefit [NNTB] 6.7).^3^ The CI for the absolute risk difference is fairly intuitive: physiotherapy might improve recovery in as many as 15% or might worsen it up to 3%. However, the CI for the NNT is trickier: physiotherapy might improve recovery in as few as 6.7 patients or worsen recovery in as few as 33.3 patients.

## IS NNT BETTER THAN THE ALTERNATIVES?

There is evidence that lay people have trouble interpreting NNT and an alternative should be used in this context.^4^ Among doctors, studies have not demonstrated that NNT is any better understood than absolute risk reductions.^5^

The supposed benefit of NNT, that it expresses a probability in terms of a number of patients, can easily be applied directly to the absolute risk difference. For example, an absolute risk reduction of 5% could be expressed as ‘five fewer adverse outcomes per 100 people treated’. This also reminds the reader that we have not been told the baseline risk: ‘five fewer than what?’

Ultimately, deciding which statistic is most easily understood is highly subjective and any measure that improves understanding is valuable. NNTs can be one useful measure, but some caution is needed, especially around the strength of evidence underlying them.
